# Widespread scope for coral adaptation under combined ocean warming and acidification

**DOI:** 10.1098/rspb.2024.1161

**Published:** 2024-09-25

**Authors:** Christopher P. Jury, Robert J. Toonen

**Affiliations:** ^1^ Hawai‘i Institute of Marine Biology, School of Ocean and Earth Science and Technology, University of Hawai‘i at Mānoa, Honolulu, HI 96744, USA

**Keywords:** coral reef, climate change, ocean acidification, evolution, heritability, adaptation

## Abstract

Reef-building coral populations are at serious risk of collapse due to the combined effects of ocean warming and acidification. Nonetheless, many corals show potential to adapt to the changing ocean conditions. Here we examine the broad sense heritability (H^2^) of coral calcification rates across an ecologically and phylogenetically diverse sampling of eight of the primary reef-building corals across the Indo-Pacific. We show that all eight species exhibit relatively high heritability of calcification rates under combined warming and acidification (0.23–0.56). Furthermore, tolerance to each factor is positively correlated and the two factors do not interact in most of the species, contrary to the idea of trade-offs between temperature and pH sensitivity, and all eight species can co-evolve tolerance to elevated temperature and reduced pH. Using these values together with historical data, we estimate potential increases in thermal tolerance of 1.0–1.7°C over the next 50 years, depending on species. None of these species are probably capable of keeping up with a high global change scenario and climate change mitigation is essential if reefs are to persist. Such estimates are critical for our understanding of how corals may respond to global change, accurately parametrizing modelled responses, and predicting rapid evolution.

## 1. Background

Coral reef ecosystems and reef-building coral populations are at serious risk of collapse within the next few decades due to ocean warming and acidification [1–4]. Ocean warming can lead to coral bleaching, reduced growth, interruptions in reproduction, increased susceptibility to disease, and mortality [5], whereas acidification often results in lower calcification [6–9] and may reduce coral recruitment [10,11]. Projected changes in coral cover on reefs as well as the potential for coral persistence under future, global change rely on modelled responses [1,2,12–14]. Such predictions, however, are only as accurate as the parameter estimates and assumptions included in the models [15,16]. Most parameter estimates are based on a few species examined in the laboratory over relatively short timescales (days to weeks) and may not reflect the longer-term responses of these organisms under a changing climate [17–19]. While critical to our understanding, short-term studies may underestimate realistic levels of coral adaptation over decadal timescales and therefore may lead to more severe projected outcomes than should be expected. The scope for coral adaptation to warming and acidification (and especially the combination of these two factors simultaneously) remains largely unknown, as does the extent to which variation in warming and acidification is heritable.

The phenotype of an organism depends on both heritable and environmental influences, but only heritable variation can be passed to offspring and acted upon by selection. Hence, the heritability of a trait provides a critical predictor of the evolutionary potential of that species or population when experiencing selective pressure. Heat stress and ocean acidification both tend to reduce coral calcification rates, though responses to these stressors vary substantially among taxa [20] as well as among individuals within species [7,8,21,22]. Calcification appears to be intimately tied to coral fitness, and calcification responses under novel conditions may result in selection for phenotypes that better match the new environment [7,8,22]. Recent studies have shown that many corals exhibit surprisingly high heritability in a number of key traits under thermal stress [21,22], and our recent work demonstrates that the heritability of calcification rates under acidification is likewise high across a diverse group of coral species [7]. Still, it is unclear if corals will be able to mount adaptive responses to warming and acidification simultaneously, and if there might be trade-offs associated with these capacities. For example, reaction norms from three Hawaiian corals reveal that *Pocillopora acuta* and *Montipora capitata* shift their relative temperature tolerances under warmer conditions, whereas *Porites compressa* shifts its relative pH tolerance under more acidic conditions, but all three react differently to dual stressors under future ocean conditions [8].

Organisms often exhibit trade-offs among different aspects of their physiological performance. Shown frequently among plants [23,24], heavy investment into particular functions or strategies typically leaves fewer resources available to engage in others, which almost certainly happens in corals as well. For example, hosting certain types of thermally tolerant algal symbionts tends to result in higher bleaching thresholds for the host coral [25,26]. These thermally tolerant symbionts, however, tend to be less productive than their more sensitive counterparts, leading to reduced coral growth rates [25,27,28]. That is, selection for one trait (thermal tolerance) comes at the expense of another trait (growth). Likewise, a single trait may exhibit contrasting responses to different stressors. For example, for some corals, increased thermal tolerance and higher survivorship under heat stress are associated with lower resistance to pathogens and reduced survivorship under that stressor [29]. Hence, adaptive responses to one environmental challenge can result in a trade-off and reduced capacity to respond to a different challenge within the same trait. If trade-offs exist between warming and acidification on coral calcification rates, then corals may show reduced capacity or inability to adapt to both factors simultaneously.

On the other hand, a common life history strategy employed by some corals, and many other organisms, is one of general stress tolerance. Stress-tolerant species or individuals may invest heavily in processes such as cellular homeostasis, tissue repair and other aspects related to stress response, thereby allowing them to survive through environmental challenges where others may not. If corals exhibit calcification trade-offs between temperature and pH tolerance, then one would expect that their responses to experimental warming and acidification should be negatively correlated between the individual stressors and/or should show a synergistic negative effect under the combined stressors. That is, tolerance to one factor should be associated with a cost and increased sensitivity to the other, or increased sensitivity under the combination of factors. Conversely, if their calcification responses to each of warming and acidification are positively correlated and do not show a synergistic negative interaction, then it would suggest that individuals within these species show variable levels of general stress tolerance, and that some individuals are especially well equipped to deal with these future ocean stressors.

Heritability can be measured in the narrow or broad sense [30,31]. Narrow sense heritability (h^2^) includes only additive genetic variation and can differ significantly from broad sense (H^2^, based on the total genetic variance) if intra-locus dominance, maternal effects, epistatic or epigenetic interactions occur [30,31]. Narrow sense heritability (h^2^) is logistically difficult to measure, particularly in most natural populations, requiring precise pedigrees over multiple generations and/or substantial genomic information [32]. Even when this substantial effort is made, estimates of heritability are notoriously underpowered and require large sample sizes to estimate with confidence [33,34]. However, broad-sense heritability (H^2^) is commonly used to predict the response to selection in plant breeding trials or human quantitative genetics where identical twin studies can be used [30,31,34,35], because they focus on the proportion of a phenotypic response in a trait that is attributable to the underlying genotypic variance [36], and the large sample sizes needed can be more easily obtained using clonal replicates of each genotype. Furthermore, complex traits controlled by many loci tend to be dominated by additive genetic variation which typically exceeds over half, and often close to 100%, of the total genetic variance, lending further value to estimation of H^2^ as the upper limit for narrow sense heritability [37]. Regardless, whether H^2^ is a precise estimation or the upper limit of coral heritability, estimating thermal and acidification tolerances of scleractinian corals and their potential to respond to increasing global temperatures and declining pH remains a critical gap in our understanding of likely responses to future ocean conditions [21].

Here, we take advantage of the ability to sample genetically distinct coral colonies (genets) to produce genetically identical clonal fragments (ramets) and use an identical twin design to estimate the broad sense heritability (H^2^) and potential for adaptation of coral calcification rates under warming, acidification and the combination of both factors. Corals of eight species were exposed to one of four treatments: (i) control (present-day temperature and pH), (ii) ocean warming (+2°C and present-day pH), (iii) ocean acidification (present-day temperature and −0.2 pH units), or (iv) combined future ocean (+2°C and −0.2 pH units). This study was conducted over 1 year, providing the corals with time to acclimatize to the treatments and helping to ensure that the measured calcification responses were a result of heritable variation among them rather than differences in their short-term histories. Furthermore, these responses were assessed in biologically diverse reef mesocosms, to ensure that the environment was as realistic as possible. These eight species are ecologically and phylogenetically diverse, representing three of the most common reef-building coral families worldwide (Acroporidae, Pocilloporidae and Poritidae), both major evolutionary lineages of scleractinian corals (Complexa and Robusta), and all four of the major coral life history strategies including three competitive species (*Montipora capitata*, *Porites compressa* and *Pocillopora meandrina*), two generalist species (*Montipora flabellata* and *M. patula*), two stress-tolerant species (*Porites evermanni* and *Porites lobata*) and one weedy species (*Pocillopora acuta*). These species, or their close relatives [38,39], include some of the most common corals across the Indo-Pacific [40], yielding broad relevance for our study. In addition to estimates of H^2^, we test for possible trade-offs associated with temperature and pH tolerance within each of these species. Finally, we calculate selection coefficients for these corals, derived from our estimates of H^2^ along with historical data and estimate potential increases in thermal tolerance for these species over the next century.

## 2. Methods

### (a) Coral collection

**Figure 1.**
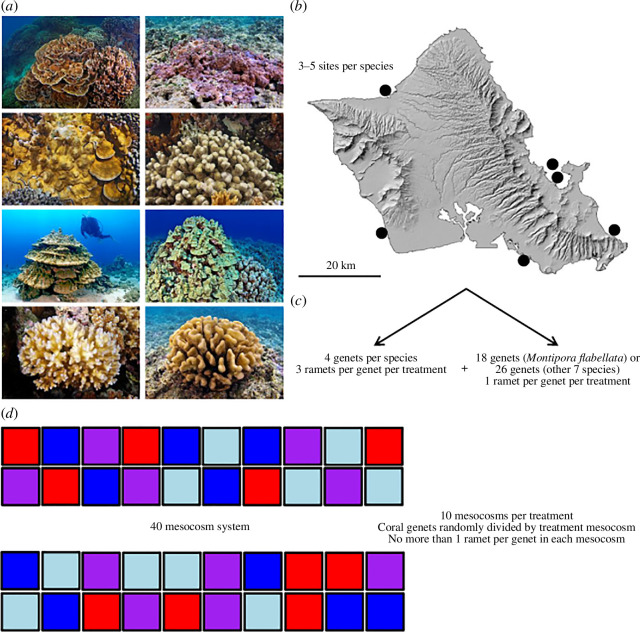
Diagram illustrating the experimental design used in this study. Representative photos of each coral are shown in (*a*) From top-left to bottom-right these are: *Montipora capitata*, *Montipora flabellata*, *Montipora patula*, *Porites compressa*, *Porites evermanni*, *Porites lobata*, *Pocillopora acuta* and *Pocillopora meandrina*. Corals were collected from a total of six locations around O‘ahu, Hawai‘i as indicated by the black dots (*b*) with each species collected from three to five of the locations, depending on local abundance. The corals were then fragmented into a series of clonal nubbins (ramets) with four genotypes (genets) per species each contributing three ramets per treatment, whereas the remaining 18 genets (*Montipora flabellata*) or 26 genets (other seven species) each contributed one ramet per genet per treatment, yielding a total of 22 genets for *Montipora flabellata* and 30 genets for the remaining seven species (*c*) The ramets were randomly allocated among mesocosms, which were themselves randomly divided among treatments, and with no more than one ramet per coral genet in each mesocosm (*d*) Mesocosms are colour-coded according to treatment: control (blue), ocean acidification (light blue), ocean warming (red) and combined future ocean (purple). Photos courtesy of Keoki Stender.

Corals were collected using a hammer and chisel at 2 ± 1 m depth from a total of six locations around O‘ahu, Hawai‘i (figure 1). Each species was collected from three to five of the sites, depending on their local abundances and sizes, for a total of 22 colonies of *Montipora flabellata* and 30 colonies (genets) of the remaining seven species. The eight species examined in this study were *Montipora capitata*, *Montipora flabellata*, *Montipora patula*, *Porites compressa*, *Porites evermanni*, *Porites lobata*, *Pocillopora acuta* and *Pocillopora meandrina*.

After collection, corals were returned to the Hawai‘i Institute of Marine Biology (HIMB) on Moku o Lo‘e (Coconut Island), fragmented into 4–12 replicate nubbins (3–5 cm coral fragments, referred to as ‘ramets’) using a diamond-coated band saw, individually attached to a labelled ceramic tile using cyanoacrylate gel, and allowed to recover for 2.5 months in a common garden under present-day average temperature for O‘ahu and ambient pH conditions, with both temperature and pH following the seasonal cycle (electronic supplementary material, figure S1).

### (b) Approach

There was a need to include both within-genet variation and among-genet variation in our estimates. Given logistical constraints about how many coral ramets could possibly be accommodated, we attempted to balance these conflicting needs by including (i) three replicate ramets from four genets for each coral species within each treatment, and (ii) one ramet per genet per treatment for the remaining genets (*n* = 22 total genets for *Montipora flabellata*; *n* = 30 for the other seven species, four of which were replicated within treatments per species). All coral ramets were randomly divided among mesocosms with no more than one ramet per genet in each mesocosm, resulting in three–four ramets per species within each mesocosm tank (figure 1).

This experiment was part of a larger mesocosm project, other components of which have been described elsewhere [41–47]. The experimental system received constant flow-through of unfiltered seawater from the adjacent reef and was initially set up with reef sand, rubble, algae, invertebrates and fish to provide a reef-like habitat (see electronic supplementary information for additional details regarding the mesocosm design). Temperature and pH of the incoming seawater were adjusted according to treatment in a series of header tanks, using aquarium heaters and CO_2_ gas injection, prior to flowing into the 70 l mesocosms at a rate of approximately 1.2 l min^−1^, for a residence time of approximately 1 h. Additional water circulation (4900 l h^−1^) was generated by seawater pumps within each mesocosm to provide water flow speeds (10–15 cm s^−1^) similar to those *in situ*. Both temperature and pH were allowed to vary according to natural daily and seasonal cycles while maintaining appropriate offsets according to treatment: control treatment (present-day temperature and pH), ocean warming treatment (+2°C and present-day pH), ocean acidification treatment (present-day temperature and −0.2 pH units), or combined future ocean treatment (+2°C and −0.2 pH units) with 10 replicate mesocosms per treatment in a 40 mesocosm system (see electronic supplementary material, figure S1). The corals were then randomly assigned to a mesocosm with either one or three replicate nubbins (ramets) per colony (genet) in each treatment, and no more than one ramet per genet in each mesocosm.

After 2.5 months of acclimatization in a common garden, temperature and pH were slowly adjusted starting on 1 February 2016 until target values were reached on 20 February 2016. The corals were then allowed 5.5 months to acclimatize to treatment conditions before calcification rates were evaluated, thereby excluding short-term history as a factor in their responses. Corals experienced heat stress during the final nine weeks of the study, during which the calcification assay was conducted (electronic supplementary material, figure S1). Calcification rates were assessed via the buoyant weighing technique [48], with initial weights taken 3–15 August 2016 (shortly after the onset of thermal stress in the heated treatments), final weights taken 26 September to 8 October 2016 (shortly after the seasonal peak in temperatures), and calcification rates were normalized to initial weight. In total, the study was conducted over nearly 1 year with approximately eight months of exposure under experimental treatment conditions, and the calcification assay was conducted over the last nine weeks of the experiment.

### (c) Coral genotyping

Multi-locus genotyping of coral hosts was performed following published methods [49,50]. Briefly, total genomic DNA was isolated using the E.Z.N.A. Tissue DNA Kit (Omega Bio-tek, Inc., Norcross, GA, USA) following the manufacturer’s protocol. Amplicons were generated via polymerase chain reaction (PCR) using microsatellite primers [51], but with short unique barcodes [52] added to each primer to identify each position in a 96-well plate. Amplicons were pooled equimolarly, and a dual-index system of adaptors was used to identify individuals on each plate and libraries were sequenced on an Illumina MiSeq platform (v. 3 2 × 300 PE) at the Hawai‘i Institute of Marine Biology. We used a custom bioinformatic genotyping workflow pipeline [49] to call alleles, which were then converted to GenoDive v. 2.0b27 [53] file format for analyses. Individual genotypes were created using two different methods. First, we used sequence length (equivalent to peak calling in a microsatellite fragment analysis *sensu* [54]), such that all sequences of the same length, regardless of underlying sequence variation, would be scored as the same allele (sequence length). Second, we identified alleles by their sequence (ID) so that only two exactly identical alleles had the same ID, whereas alleles with the same length but differing in nucleotide composition would have different allelic IDs. Similar to previous findings [49], both approaches gave the same result. Using the ‘assign clones’ feature of GenoDive [55], we tested whether coral colonies sampled in the field had a unique multi-locus genotype. To be conservative, we allowed for up to two scoring errors among individuals and checked potential clones against the location of collection.

### (d) Historical temperature tolerances

Over the last 50 years, the mean seawater temperature around O‘ahu has warmed at a rate of 1.9°C per century and acidified at a rate of 0.13 pH units per century [19,56] (but note that pH data first became available in 1992) (electronic supplementary material, figure S2). The rate of acidification will accelerate in the future as a consequence of reduced seawater buffer capacity [57]. In 1970, survivorship of corals exposed to temperatures near their upper thermal limits (31.0°C) was assessed for two of the species included in this study (*Montipora capitata* and *Pocillopora acuta*) [58]. In 2017, this study was repeated (31.4°C) [19] (data shown in the original publications). We examined changes in their temperature tolerances by fitting logistic regressions to survivorship using the R package ‘MASS’ in the following way (given differences in data overlap among datasets). For *Montipora capitata,* we assessed the LD20 (degree heating weeks, DHW, which results in 20% mortality at these upper thermal limits) and for *Pocillopora acuta,* we assessed the LD50 (DHW which result in 50% mortality at these upper thermal limits) in each of 1970 and 2017, again using ‘MASS’ to define these values. These fits resulted in the following estimates: LD20 for *Montipora capitata* of 1.30 ± 0.39 and 20.17 ± 2.46 DHW in 1970 and 2017, respectively; LD50 for *Pocillopora acuta* of 1.47 ± 0.56 and 18.68 ± 1.24 DHW in 1970 and 2017, respectively. We then estimated the change in these DHW tolerances for each species as proxies for their responses to selection (R) over the period 1970–2017, as described below.

### (e) Statistics

To examine treatment effects on calcification rates, for each species individually, an ANOVA was fit with temperature, pH and collection site as fixed factors, and coral colony (genet), header tank and mesocosm as nested factors. Due to the smaller sample size for *Montipora flabellata*, there were an insufficient number of degrees of freedom to fit the full model. Instead, we first fit a model with all factors included except coral genet. Mesocosm effects were not significant, so this factor was dropped and a second model was then fit which included genet. Model fits were assessed via diagnostic plots of the residuals and in all cases the data adequately satisfied ANOVA assumptions. A Tukey honestly significant difference (HSD) was used as a *post hoc* to examine significant treatment effects.

Broad sense heritability (H^2^) was estimated using a Bayesian modelling approach, similar to that used in previous work [59,60]. The models were fit with the R package ‘MCMCglmm’ [61] with temperature and pH as fixed effects and coral genet, collection site, header tank and mesocosm as random effects. Models were run for 100 000 iterations, storing the Markov chain every 50 iterations, and with the first 15 000 iterations used as a burn-in period. Heritability was estimated as the proportion of the phenotypic variance which was explained by genotype [59,60].

To test for possible trade-offs between temperature and pH tolerance, and the hypothesis that these tolerances are related to a general stress-tolerance strategy, the association between temperature and pH response was examined for each species using Pearson’s correlation. Temperature tolerance was calculated as the change in mean calcification rate between the control and the ocean warming treatment, whereas pH tolerance was the difference between the control and the ocean acidification treatment. Furthermore, we considered if there were interactive effects between temperature and pH on calcification rates identified by the ANOVAs, as another indicator of trade-offs in these tolerances.

For *Montipora capitata* and *Pocillopora acuta,* we estimated their responses to selection (R) based on changes in their LD20 and LD50 values, respectively, from 1970 to 2017. The error associated with these estimates was determined from Monte Carlo simulations using the R package ‘propagate’ and derived from 100 000 simulations. Along with our heritability estimates, as described above, as well as the classical (univariate) breeder’s equation, R = H^2^S, we estimate the selection coefficients for these species under the selective pressure that they have experienced over the last 50 years, where H^2^ is the broad sense heritability (including additive, dominance and epistatic variance) which represents the proportion of the selection differential (S) that can be realized as the response (R) to selection [30,36,59,62], and with the error associated with S propagated in the same way. Again, we assume that broad sense heritability (H^2^) provides an upper bound for narrow sense heritability (h^2^), though the two values are probably similar if these traits are influenced by many genetic loci. Selection differentials for the remaining six species are unknown, so to be conservative we assumed that they experience selection similar to that for *Montipora capitata* (the lower of the two selection coefficients) and estimated their responses to selection (R) based on these assumed values and their measured heritabilities, with uncertainties propagated as above.

Analyses were performed in R v. 4.0.3 [63].

### (f) Effects of unbalanced sampling design

We considered how an unbalanced sampling design might affect our estimates. In particular, our study was slightly unbalanced because most of the coral genets contributed one ramet per treatment whereas four genets per species contributed three ramets per treatment. In addition, a small percentage of the ramets died during the acclimatization phase (18 nubbins, or 1.5% of the total, and affecting 12 of the 232 genets) resulting in representation of 87–100% of the genets across all four treatments, depending on species.

For the ANOVAs, this slight unbalance has very little effect. ANOVA is highly robust to modest discrepancies in sample size and missing observations, such as occur in this study. With the heritability estimates, modelling work [36,62] suggests that our sampling design results in a less than 3% additional uncertainty in H^2^ for *Pocillopora acuta* and *Pocillopora meandrina*, and far less among the other species. This small uncertainty yields only a trivial effect on our estimates of selection coefficients (S) or responses to selection (R).

## 3. Results

### (a) Coral collection and genotyping

Depending on local abundances, each coral species was sampled from three to five of the six collection locations around O‘ahu resulting in 22 parent colonies for *Montipora flabellata* and 30 parent colonies for each of the remaining seven species (figure 1, electronic supplementary material, table S1). Each of the 232 parent colonies samples exhibited a unique multi-locus genotype [42,64]. To be conservative, we allowed for up to two scoring errors in these microsatellite analyses which returned only a single potential clonal pair among these corals (*Porites compressa* colonies #1 and #3 from Waimānalo). These two corals, however, displayed highly distinctive coloration (yellow-grey vs. tan) and morphology (smoother vs. knobbier branches) which they maintained for a year while growing in a common garden with the other colony. This gave us confidence that none of the coral colonies sampled for this study were clonally derived and that each colony represents a distinct genet.

### (b) Environmental conditions

We exposed the corals to the target treatment conditions, consisting of present-day mean temperature and pH as well as warming of +2°C and acidification of −0.2 pH units while preserving the natural daily and seasonal fluctuations of these parameters (electronic supplementary material, figure S1). In the heated treatments (ocean warming and combined future ocean) the corals accumulated approximately 14 DHW during the calcification assay.

### (c) Coral survivorship

A small number of coral nubbins (18 ramets, or 1.5% of the 1184 ramets total) died during the acclimatization phase. Mortality was restricted primarily to one genet of *Pocillopora meandrina*, wherein 7 of the 12 ramets died. In addition, one ramet each from 11 of the 232 coral genets also died. These dead nubbins were excluded from the analyses. During the nine-week heat stress event, when the calcification assay was being conducted, an additional 14 nubbins (1.2% of the total) also died, but these ramets were included in the analyses to avoid biasing the data against the most thermally sensitive individuals.

### (d) Treatment effects on calcification rates

**Figure 2.**
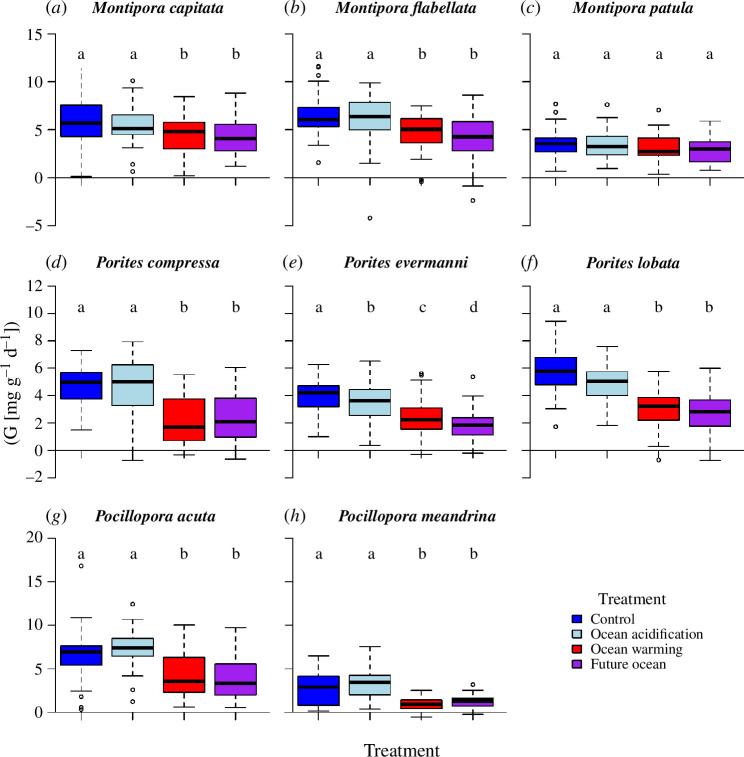
Boxplot of treatment effects on coral calcification rate (G) for each species. All eight species experienced reduced calcification under warming. Three of the species (*Montipora flabellata*, *Porites evermanni* and *Porites lobata*) exhibited reduced calcification under acidification, whereas *Pocillopora meandrina* showed increased calcification, and the other four species did not respond significantly to reduced pH. For most of the species, temperature and pH effects were additive, whereas for *Pocillopora meandrina* there was a significant Temp × pH interaction such that the resistance of this species to acidification was diminished under elevated temperature. Horizontal black bar is the median, box edges are 75% confidence intervals, box whiskers are 95% confidence intervals, and points show outliers. Non-bolded letters above each box indicate Tukey HSD post hoc test results for pairwise contrasts among treatments; groups sharing a letter within each panel are not significantly different at *α* = 0.05. *n* = 22 genotypes (genets) for *Montipora flabellata* and *n* = 30 genets for the remaining seven species. Note differences in scale among rows.

For all eight species, there were significant main effects of temperature on calcification rates, with all eight species experiencing reduced calcification at elevated temperature (figure 2, electronic supplementary material, table S2). In addition, five of the species also experienced a significant main effect of acidification, and calcification tended to decrease under reduced pH for *Montipora capitata*, *Montipora flabellata*, *Porites evermanni* and *Porites lobata*, yet increased for *Pocillopora meandrina*. Only one species, *Pocillopora meandrina*, exhibited a significant Temp × pH interaction, because the resistance of this species to acidification was diminished under heating. As is typical for most corals, linear extension rates ranged from approximately 1 to 10 cm yr^− 1^, depending on species and genet.

### (e) Heritability

**Figure 3.**
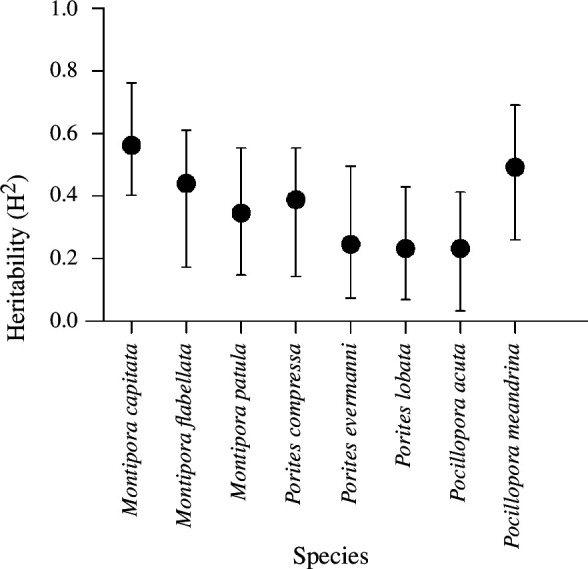
Broad sense heritability (H*^2^*) for each coral species under combined warming and acidification. Points show best estimates and error bars are 95% confidence intervals. *n* = 22 genotypes (genets) for *Montipora flabellata* and *n* = 30 genets for the remaining seven species.

Estimates of heritability (H^2^) ranged from a low of 0.23 in *Pocillopora acuta* to a high of 0.56 in *Montipora capitata*, and calcification rates were significantly heritable for all eight species (figure 3). See table 1 for a summary of major findings from this study according to species.

**Table 1.** Summary of major findings from this study according to species. Columns show treatment effects on coral calcification due to heating (temp effects), acidification (pH effects), interactive effects between factors (temp × pH interaction) and estimates of broad sense heritability (H^2^).

species	family	life history strategy	temp effects	pH effects	temp × pH interaction	H^2^
*Montipora capitata*	Acroporidae	competitive	negative	negative	no	0.56
*Montipora flabellata*	Acroporidae	generalist	negative	negative	no	0.44
*Montipora patula*	Acroporidae	generalist	negative	neutral	no	0.34
*Porites compressa*	Poritidae	competitive	negative	neutral	no	0.39
*Porites evermanni*	Poritidae	stress tolerant	negative	negative	no	0.24
*Porites lobata*	Poritidae	stress tolerant	negative	negative	no	0.23
*Pocillopora actua*	Pocilloporidae	weedy	negative	neutral	no	0.23
*Pocillopora meandrina*	Pocilloporidae	competitive	negative	positive	yes	0.49

### (f) Temperature and pH tolerance correlations

**Figure 4.**
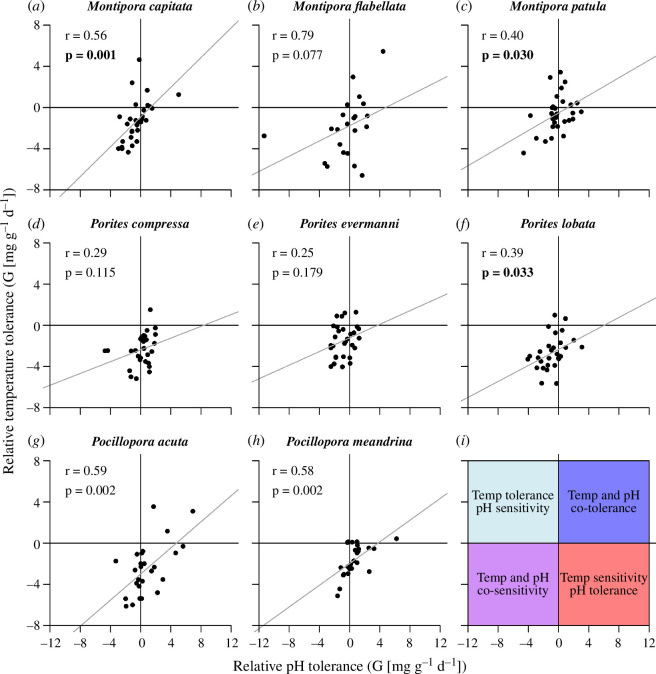
Scatterplot of relative pH and temperature tolerances for each coral species. Relative pH tolerance is defined as the change in calcification rate in the ocean acidification treatment relative to the control treatment whereas relative temperature tolerance is defined as the change in calcification rate in the ocean warming treatment relative to the control treatment for each species (*a–h*) Grey line in each plot is a linear regression of the relationship between pH and temperature tolerance for each species. The plot in the lower right (*i*) is a visual guide to aid in interpretation of the panels. Correlation coefficients (*r*) and *p*-values for the regressions are shown in each panel; significant *p*-values at *α* = 0.05 are shown in bold. Five of the species (*Montipora capitata*, *Montipora patula*, *Porites lobata*, *Pocillopora acuta* and *Pocillopora meandrina*) show significant positive associations between pH and temperature tolerance, whereas the other three species (*Montipora flabellata*, *Porites compressa* and *Porites evermanni*) exhibit similar, non-significant trends. This analysis tests for possible calcification trade-offs between pH and temperature tolerance. Only *Pocillopora meandrina* exhibited a significant Temp × pH interaction in the combined future ocean treatment, which is likely to reduce the capacity of this species to adapt to the combined stressors. All eight species are expected to be capable of adapting to ocean warming, ocean acidification and the combination of both factors, though *Pocillopora meandrina* is likely to have diminished capacity to respond to the combination as compared with each factor individually. Due to mortality of a few coral ramets not all genets could be included in the analysis. *n* = 22 genets for *Montipora flabellata*, *n* = 26 for *Pocillopora acuta* and *Pocillopora meandrina*, and *n* = 30 for the remaining five species.

We define relative temperature tolerance as the change in calcification in the ocean warming treatment compared with the control, and relative acidification tolerance as the change in calcification in the ocean acidification treatment as compared with the control. Five of the species (*Montipora capitata*, *Montipora patula*, *Porites lobata*, *Pocillopora acuta* and *Pocillopora meandrina*) exhibited a significant positive correlation between temperature and pH tolerance, whereas the remaining three species (*Montipora flabellata*, *Porites compressa* and *Porites evermanni*) showed similar, non-significant trends (figure 4, electronic supplementary material, table S3). None of the corals exhibited negative correlations between temperature and acidification tolerance, as would be expected if there were trade-offs between these tolerances.

### (g) Responses to selection

**Figure 5.**
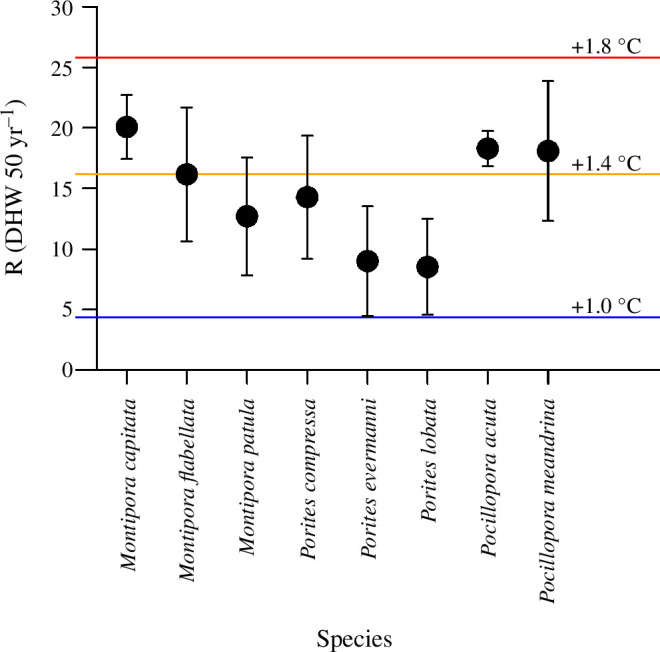
Estimated responses to selection for each species over the next 50 years. For *Montipora capitata* and *Pocillopora acuta,* values were empirically derived based on observed change in DHW tolerance between 1970 and 2017. For the remaining species, the values were estimated based on the heritability values derived here and assuming a selection coefficient similar to that for *Montipora capitata*. Horizontal lines show the mean annual DHW accumulation at warming levels of 1.0, 1.4, and 1.8°C, based on NOAA data for the main Hawaiian islands.

We project that all eight species are capable of evolving an increase in thermal tolerance over the next 50 years. These values ranged from a low of 8.5 DHW in *Porites lobata* to a high of 20.0 DHW in *Montipora capitata* (figure 5).

## 4. Discussion

The scope for corals to adapt to combined warming and acidification will play a key role in their responses to global change over coming decades [4,9,12,15,16,18,48]. While studies have sometimes assumed that corals exhibit low evolutionary potential and therefore the responses of future generations will mirror those of today [1,2,4,65,66], there is mounting evidence that many coral species harbour greater capacity to adapt to the changing climate than is often appreciated [8,19,21,22]. Because of the imminent threat that ocean warming poses to corals, most studies examining their capacity to adapt or acclimatize to novel conditions have focused on heat tolerance [21,22]. In contrast, and in spite of a rich literature characterizing coral responses to ocean acidification [6,9,18], far less is known about their capacity to adapt to reduced pH, and very few studies have examined their capacity to adapt to the combination of these two factors [59,60].

Corals often experience reduced calcification rates under acidification [9,18,48,65], but many of the species in this study showed comparatively small responses to reduced pH. Coral responses to acidification, however, are not necessarily linear [8,67–69]. Indeed, three of these species (*Montipora capitata*, *Pocillopora acuta* and *Porites compressa*) tend to achieve higher calcification rates under modest acidification (−0.15 pH units) [8] yet experience reduced calcification under more severe pH reductions (−0.3 to −0.4 pH units) [7], and all eight species tend to experience reduced calcification under severe acidification (−0.4 pH units) [7]. Coral responses to acidification, however, are complex and the mechanisms that govern them are not yet fully understood [9,18,70,71]. Under acidification, corals probably benefit from increased bicarbonate supply yet also suffer due to increased proton concentration [9,70]. The −0.2 pH unit change included in this study may roughly split the difference between the positive effects of carbon enrichment and the negative effects of higher proton concentration on calcification rates for many of these species.

None of the eight coral species examined here exhibited significant negative correlations between temperature and pH tolerance, and only one of the species (*Pocillopora meandrina*) exhibited a significant Temp × pH interaction, because its pH resistance was diminished under heating. Hence, none showed calcification responses consistent with there being clear trade-offs in their sensitivity to ocean warming and acidification and all eight species appear to be capable of adapting to each factor independently as well as the combination of both factors. For *Pocillopora meandrina*, however, the significant Temp × pH interaction suggests that this species has lower capacity to adapt to combined warming and acidification than it does to either factor by itself. If, however, there were no cost to maintaining comparatively high temperature or pH tolerance then these traits should have already gone to fixation within the populations, in which case we would have found very low heritability of this trait. Given the comparatively high heritability in all species, trade-offs may exist with unmeasured variables such as tolerance to other important environmental characteristics such as resistance to wave energy, reproductive output or disease tolerance. Rather, corals often show parabolic performance curves over a range of temperature and pH conditions [8,67–69], and there is no reason to think that all corals are best adapted to a particular set of conditions. Instead, these data suggest that coral individuals within populations exhibit a range of environmental tolerances and some corals are inherently better adapted to certain environments than are others.

In contrast to trade-offs, most of the species showed a significant positive correlation between temperature and pH tolerance. These responses are consistent with the hypothesis that sensitivity to both warming and acidification is related to general stress tolerance among colonies within these species. If that is the case, then these stress-tolerant individuals may also show higher performance under other sorts of environmental insults. Indeed, Wright *et al*. [59] found that tolerance to heat stress, acidification and pathogen exposure all tend to be positively correlated in an Australian coral. Furthermore, this relationship suggests that pH and temperature tolerance can co-evolve in all eight species.

The opportunity for adaptive change of populations in response to natural or artificial selection relies on the genetic variance underlying phenotypic traits, and predictive models of adaptation to global change require estimates of the proportion of that trait variance which is explained by heritable genetic factors [30,31]. This explanative proportion is most accurately estimated by narrow sense heritability (h^2^), which depends strictly on additive genetic variance and provides the fodder for natural selection [33]. Such studies, however, require enormous investment of resources to produce precise pedigrees or substantial genomic information [34]. In contrast, broad sense heritability (H^2^), as we measure here, is easier and faster to estimate, but also includes other heritable factors, such as dominance, epistatic, maternal and epigenetic effects. Thus, H^2^ provides an upper bound for narrow sense (h^2^) heritability [30,31,72]. However, both theory and data demonstrate that non-additive interactions at the level of individual genes are unlikely to greatly impact variance for complex traits controlled by many genes, such as thermal and acidification tolerances, and often close to 100% of the total observed variation is additive [37]. Despite the importance of heritability estimates, they remain elusive for scleractinian corals [21]. Thus, broad sense heritability (H^2^) provides a reasonable first pass at estimating the underlying genetic variance for coral thermal and pH tolerance, and at worst, provides an upper bound for the value. Here we show that the broad sense heritability of coral calcification rates under combined ocean warming and acidification is fairly high, ranging from 0.23 to 0.56, and consistent with other organisms in which life history traits are linked directly to fitness [73–75]. These values are also consistent with previous reports examining the heritability of scleractinian calcification under warming alone (H^2^ approx. 0.25–0.5) [21] or acidification alone (H^2^ approx. 0.32–0.61) [7]. A useful parameter is derived from the classical breeder’s equation, R = h^2^S, where R is the response to selection, h^2^ is the narrow sense heritability and S is the selection coefficient. Given caveats such as the fact that most coral populations are very large [76], the breeder’s equation provides a useful metric for our understanding of how coral populations may evolve in the future. While it is commonly assumed that corals are under strong selective pressure for higher temperature tolerances, given their recent and predicted future declines due to heating [1–4], and that they might be under selective pressure due to acidification [7], the paucity of quantitative data for selection coefficients contributes uncertainty to model predictions regarding coral responses to global change and the potential for adaptation.

Here, we empirically estimate not only the broad sense heritability (H^2^) of coral calcification rates under combined warming and acidification but also the selection coefficients (S) and responses to selection (R) for two of the species (*Montipora capitata* and *Pocillopora acuta*), derived from historical data, and assuming that h^2^ and H^2^ are similar. It is important to note that these selection coefficients represent the average strength of selection over the period 1970–2017. Without a doubt, both selection and responses to selection will vary over space and time depending on the environmental characteristics that coral populations experience. Given these caveats, and assuming that the remaining six coral species exhibit similar selection coefficients, we estimate that these coral species can probably increase their thermal tolerances by a mean of 8.5 DHW (*Porites lobata*) to 20.0 DHW (*Montipora capitata*) over the next 50 years. Considering the uncertainties in these estimates, these values correspond to an increase of approximately 1.0–1.7°C, depending on species. Under a high CO_2_ emissions scenario [77], none of these species are probably capable of keeping up with the greater than 3°C of warming expected by the end of the century. In contrast, if climate change is limited to no more than 2°C above the pre-industrial (approx. 0.8°C above present-day), in line with Paris Climate Agreement targets [78], then all of these species might be able to persist, albeit very likely with changes in coral reef community structure [47,64]. Hence, while these data show that diverse coral taxa possess heritable variation with clear capacity to respond to selection for both ocean warming and acidification, substantial climate change mitigation efforts remain essential if coral reefs are to persist over the twenty-first century and beyond.

## Data Availability

Data and code are freely accessible through Dryad [79]. Supplementary material is available online [80].
